# Host-Driven Phosphorylation Appears to Regulate the Budding Activity of the Lassa Virus Matrix Protein

**DOI:** 10.3390/pathogens7040097

**Published:** 2018-12-09

**Authors:** Christopher M. Ziegler, Philip Eisenhauer, Inessa Manuelyan, Marion E. Weir, Emily A. Bruce, Bryan A. Ballif, Jason Botten

**Affiliations:** 1 Department of Medicine, Division of Immunobiology, University of Vermont, Burlington, VT 05405, USA; cziegler@uvm.edu (C.M.Z.); philip.eisenhauer@med.uvm.edu (P.E.); Inessa.Manuelyan@uvm.edu (I.M.); Emily.Bruce@med.uvm.edu (E.A.B.); 2 Cellular, Molecular and Biomedical Sciences Graduate Program, University of Vermont, Burlington, VT 05405, USA; 3 Department of Biology, University of Vermont, Burlington, VT 05405, USA; marion.weir@cellsignal.com (M.E.W.); bballif@uvm.edu (B.A.B.); 4 Department of Microbiology and Molecular Genetics, University of Vermont, Burlington, VT 05405, USA

**Keywords:** Lassa virus, Z protein, late domain, PPXY, budding, release, matrix protein, phosphorylation, arenavirus, mass spectrometry

## Abstract

Lassa mammarenavirus (LASV) is an enveloped RNA virus that can cause Lassa fever, an acute hemorrhagic fever syndrome associated with significant morbidity and high rates of fatality in endemic regions of western Africa. The arenavirus matrix protein Z has several functions during the virus life cycle, including coordinating viral assembly, driving the release of new virus particles, regulating viral polymerase activity, and antagonizing the host antiviral response. There is limited knowledge regarding how the various functions of Z are regulated. To investigate possible means of regulation, mass spectrometry was used to identify potential sites of phosphorylation in the LASV Z protein. This analysis revealed that two serines (S18, S98) and one tyrosine (Y97) are phosphorylated in the flexible N- and C-terminal regions of the protein. Notably, two of these sites, Y97 and S98, are located in (Y97) or directly adjacent to (S98) the PPXY late domain, an important motif for virus release. Studies with non-phosphorylatable and phosphomimetic Z proteins revealed that these sites are important regulators of the release of LASV particles and that host-driven, reversible phosphorylation may play an important role in the regulation of LASV Z protein function.

## 1. Introduction

The *Mammarenavirus* genus is comprised primarily of rodent-borne viruses, several of which are capable of causing severe hemorrhagic fever syndromes in humans [1]. Lassa virus (LASV), the causative agent of Lassa fever, is carried primarily by the multimammate rat, *Mastomys natalensis*, but other carrier rodents have been identified recently [2,3,4]. LASV infects up to an estimated 300,000 people each year in western Africa following exposure to rodent excreta or through hunting and consumption of infected rats [2,5,6,7,8]. Lassa virus can also spread person-to-person through direct contact with infected bodily fluids, and this pattern of transmission has occurred repeatedly in hospital workers caring for Lassa fever patients since its discovery in 1969 [9,10,11]. Overall, the case fatality rate is estimated at 1–2%, but significantly higher rates of fatality occur in hospitalized patients [5]. Since 2008, outbreaks in Sierra Leone have resulted in an overall case fatality rate of 69% in hospitalized patients [12]. LASV infection results in fetal loss in most cases [13]. Further, pregnancy greatly increases the risk of fatality from Lassa fever for the mother [13]. Of Lassa fever survivors who were hospitalized, approximately one-third developed hearing loss, and in two-thirds of those patients, the hearing deficit was permanent [14]. Intravenous ribavirin treatment has been shown to reduce mortality from Lassa fever, particularly if administered during the first six days of fever onset, but there is a clear need for more effective therapies [5]. No United States Food and Drug Administration (FDA)-approved vaccines exist for the prevention of LASV infection.

Arenaviruses have a simple, negative-strand RNA genome that encodes a total of four proteins on two segments. The small (S) segment encodes both the nucleoprotein (NP), which encapsidates the viral genome and is required for its replication, and the envelope glycoprotein (GP), which interacts with cell surface receptors to mediate cell entry and membrane fusion [15,16,17,18,19,20]. The large (L) genome segment encodes the viral RNA-dependent RNA polymerase (L), which replicates and transcribes the genome, and the viral matrix protein (Z) [19,20,21,22]. The Z protein is a structural component of the virion, forming a matrix layer on the inner leaflet of the viral envelope, and carries out an array of important functions during viral propagation [23,24]. The Z protein of pathogenic arenaviruses antagonizes interferon production by binding to retinoic acid-inducible gene I (RIG-I) and melanoma differentiation-associated protein 5 (MDA5) to disrupt interactions between RIG-I-like receptors and mitochondrial antiviral signaling (MAVS) [25]. Z can inhibit the translation of capped cellular mRNAs and also regulate the activity of the viral polymerase [26,27,28]. Z is also an important coordinator of virus particle assembly by interacting with each of the other three viral proteins [29,30].

In addition to these functions, the Z protein is both necessary and sufficient for driving the efficient release of nascent virus particles [23,31,32]. However, minimal levels of virus can be recovered without Z [33]. Two major motifs in the Z protein mediate the efficient release of virus particles. First, a myristoylation modification of the second residue in Z, a glycine, mediates Z’s interaction with cellular membranes, a requirement for efficient release [34,35]. Second, all arenavirus Z proteins, with the exception of the Tacaribe virus Z protein, contain one or two proline-rich, late domains near the C-terminus [23,32]. These viral late domains are presumably responsible for recruiting the cellular endosomal sorting complex required for transport (ESCRT) pathway to mediate membrane scission, which is the final step in the virus budding process [36]. New World arenavirus Z proteins contain a P(S/T)AP-type late domain, which can bind the ESCRT-I protein Tsg101, while the Old World Z proteins of the lymphocytic choriomeningitis virus (LCMV) and Dandenong virus contain only a PPXY late domain, which can bind Nedd4-family E3 ubiquitin ligases [23,24,32,37]. Additionally, the Z proteins of several Old World arenaviruses, including Lassa, Mobala, Mopeia, and Ippy viruses, encode both the PPXY and the P(S/T)AP late domains [23,24,32]. In the case of LASV, decreased virus-like particle (VLP) release occurs following mutation of either of LASV Z’s PTAP and PPXY late domains, disruption of Z’s recruitment of Nedd4-family proteins, and/or loss of specific ESCRT components [23,24,32,37,38,39]. The release of VLPs for the related Old World arenavirus LCMV is similarly impacted by PPXY late domain or ESCRT component disruption [32]. However, recent work from our lab demonstrated that in a whole virus system, the sole late domain in the LCMV Z protein and the ESCRT pathway it recruits were specifically required for the production of defective interfering particles but not of infectious virus [40]. This illustrates the complexity and diversity in arenavirus release and highlights the need for a greater understanding of the factors involved in this process.

Protein phosphorylation is a reversible post-translational modification that can regulate the activity of enzymes, mediate protein–protein interactions, alter the subcellular localization, conformation, and/or oligomeric state of proteins, as well as regulate the addition or removal of other types of post-translation modifications [41]. This important type of modification is not limited to cellular proteins; a growing body of literature has demonstrated that phosphorylation of viral proteins has important consequences for different viral processes [42,43,44,45]. Phosphorylation sites of functional significance have been identified on the matrix proteins in an array of RNA viruses, including the Z protein of the Old World arenavirus LCMV [40,46,47,48,49,50,51]. Accordingly, we hypothesized that the functionality of the LASV Z protein may be regulated by phosphorylation. To address this hypothesis, mass spectrometry was used to identify phosphorylated residues in LASV Z. This approach revealed three sites of phosphorylation, including two phosphorylated serines and one tyrosine. A VLP release assay demonstrated that serine phosphorylation may negatively impact virus release. These findings are an important first step toward understanding how various functions of the LASV Z protein can be regulated by post-translational modifications.

## 2. Results and Discussion

**Figure 1:**
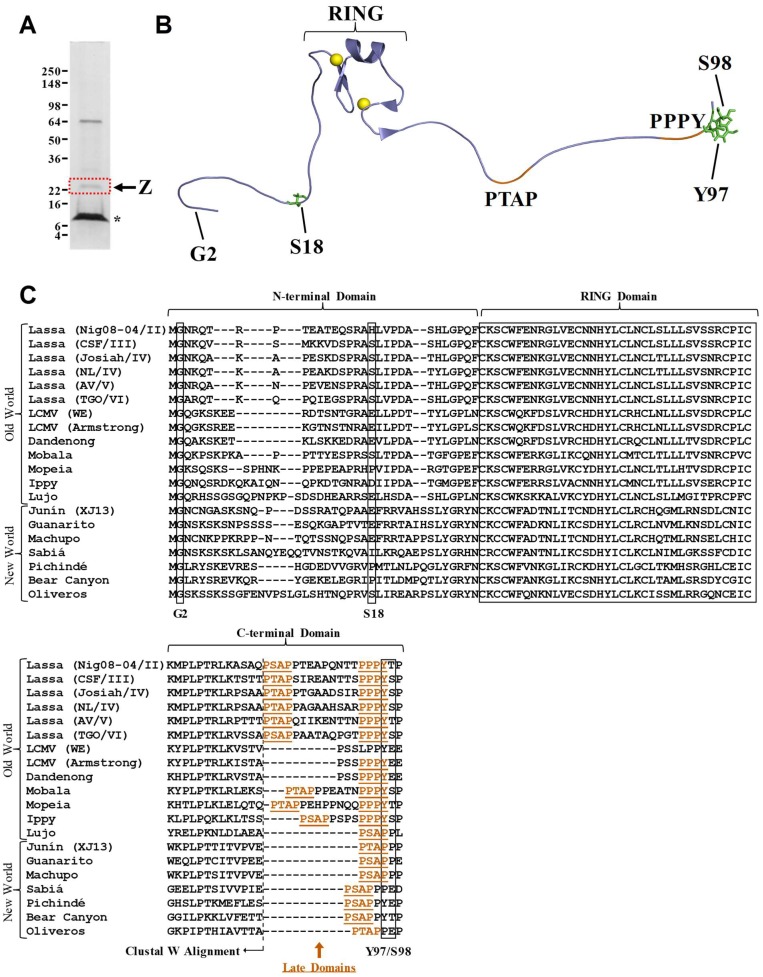
Identification of phosphorylation sites in the Lassa mammarenavirus (LASV) Z matrix protein. (**A**) Coomassie-stained polyacrylamide gel of affinity-purified LASV Z. Streptavidin-coated magnetic beads were used to affinity purify Z from virus-like particles (VLPs) released from cells co-transfected with plasmids encoding a biotin ligase and LASV Z C-terminally tagged with a biotin acceptor peptide (BAP). The band corresponding to the LASV Z protein (indicated by the red box) was excised from the gel and subjected to proteolytic digestion with trypsin or a combination of trypsin and chymotrypsin. The resultant peptides were extracted and subjected to liquid chromatography–tandem mass spectrometry analysis. The asterisk denotes the band of monomeric streptavidin that is eluted from streptavidin beads following boiling. (**B**) The protein nuclear magnetic resonance structure of the Josiah strain of the LASV Z matrix (PDB 2M1S) is shown. The side chains of the phosphorylated residues (S18, Y97, and S98) are highlighted in green on the protein structure. The two late domains found in LASV Z, PTAP and PPPY, are colored orange. Zinc ions, which are coordinated by the central really interesting new gene (RING) domain of Z, are shown as yellow spheres in the protein structure. The myristoylated glycine residue (at position 2) is also indicated. (**C**) Protein sequence alignment of select mammarenavirus Z proteins. The Clustal Omega multiple sequence alignment tool was used to align the sequences of selected mammarenavirus Z proteins. The portion of the C-terminal region of each Z protein (the amino acids to the right of the dashed vertical line) containing the late domains (designated as orange, underlined amino acids) was aligned with LASV Z strain Josiah, starting with the most C-terminal amino acid. The following accession numbers were used: GU481069.1 (Lassa mammarenavirus, strain Nig08-04), AAO59514.1 (Lassa mammarenavirus, strain CSF), NP_694871.1 (Lassa mammarenavirus, strain Josiah), AAO59510.1 (Lassa mammarenavirus, strain NL), AAO59508.1 (Lassa mammarenavirus, strain AV), MF990887.1 (Lassa mammarenavirus, strain TGO) AAD03395.1 (Lymphocytic choriomeningitis mammarenavirus, strain WE), ABC96003.1 (Lymphocytic choriomeningitis mammarenavirus, strain Armstrong 53b), ABY20731.1 (Dandenong virus), ABC71138.1 (Mobala mammarenavirus), ABC71136.1 (Mopeia mammarenavirus, strain Mozambique), ABC71142.1 (Ippy mammarenavirus), YP_002929492.1 (Lujo mammarenavirus), NP_899216.1 (Junín mammarenavirus, strain XJ13), NP_899220.1 (Guanarito mammarenavirus), NP_899214.1 (Machupo mammarenavirus), ABY59837.1 (Brazilian mammarenavirus), YP_138535.1 (Pichindé mammarenavirus), YP_001649224.1 (Bear Canyon mammarenavirus), YP_001649215.1 (Oliveros mammarenavirus). For each LASV isolate, the corresponding lineage is listed after the strain.

To identify sites of phosphorylation, LASV Z protein was affinity purified from VLPs produced from plasmid-transfected cells and subjected to protein gel electrophoresis (Figure 1A). The gel band corresponding to LASV Z was excised and proteolytically digested. The resulting peptides were analyzed by liquid chromatography–tandem mass spectrometry. Three phosphorylated residues in the LASV Z protein were identified: two serine residues (S18 and S98) and one tyrosine residue (Y97) (Figure 1B, Figures S1 and S2). Representative mass spectra and the accompanying fragment ion tables for peptides harboring phosphorylated Y97 (Figure S1A), phosphorylated S98 (Figure S1C), as well as corresponding unphosphorylated peptides are shown (Figure S1B). Representative mass spectra and corresponding fragment ion tables of the peptides harboring phosphorylated S18 (Figure S2A) and unphosphorylated S18 (Figure S2B) are shown.

Each phosphorylation site was mapped onto the NMR structure of LASV Z (Figure 1B) [52]. The arenavirus Z protein is comprised of a central zinc-binding, really interesting new gene (RING) domain flanked by N- and C-terminal domains that appear to be flexible and relatively unstructured [52,53,54,55,56,57]. All three phosphorylation sites identified are located in these flexible N-terminal (S18) or C-terminal (Y97 and S98) domains of the Z protein (Figure 1B,C) [52]. The N-terminal domain of Z is known to mediate several important functions. It contains a myristoylation site at the second glycine residue which is required for virus budding as well as Z’s interaction with the plasma membrane and with the envelope glycoprotein [30,34,35]. Other conserved residues in the N-terminal domain contribute to the production of infectious virus-like particles [58]. Additionally, the N-terminal domain of pathogenic arenaviruses, including LASV, but not of non-pathogenic arenaviruses, can directly bind to and inhibit the function of retinoic acid-inducible gene 1-like receptors (RLRs), which results in decreased macrophage activation [25,59]. The Clustal Omega multiple sequence alignment tool was used to align select mammarenavirus Z proteins (Figure 1C) [60]. This revealed that the S18 phosphorylation site is conserved across LASV strains representing lineages III–VI, as well as in Mobala virus and the New World Oliveros virus (Figure 1C) [61,62,63]. Notably, a histidine residue is found at this position in multiple lineage II LASV isolates that have recently been sequenced [64]. Interestingly, aspartic acid and glutamic acid, which are negatively charged at a physiological pH similar to phosphate, are substituted for S18 in a number of other New and Old World arenavirus Z proteins, suggesting that a negative charge at this position may be important for Z functionality (Figure 1C).

The Y97 and S98 phosphorylation sites lie within the C-terminal tail of the LASV Z protein, a region which contains two proline-rich late domains, PTAP and PPPY (Figure 1B,C). The C-terminal amino acids containing the late domains of selected mammarenavirus Z proteins were aligned (Figure 1C). This analysis showed that Y97 is conserved across the majority of Old World mammarenaviruses (except for Lujo virus) and aligns with Pichindé and Bear Canyon New World mammarenaviruses (Figure 1C). Conservation of a serine or threonine at residues that align with LASV Z S98 is fairly common among Old World mammarenaviruses (Figure 1C). Notably, LCMV possesses a glutamic acid at this position which could substitute for the negative charge of a phosphorylated serine (Figure 1C). However, LASV Z S98 is directly followed by a proline residue. Interestingly, phosphorylated serine or threonine residues directly preceding a proline (pS/T–P) constitute a type of motif that can be recognized by class IV WW domains, including the peptidylprolyl cis/trans isomerase, NIMA-interacting 1 protein (Pin1) [65,66]. Pin1 can mediate conformational changes in substrate proteins that result in altered protein stability or phosphorylation state [67]. Because Pin1 has been implicated in different viral infections and has been shown to bind pS/T–P motifs within viral proteins from hepatitis B virus [68], human T-cell leukemia virus type 1 [69,70], human immunodeficiency virus 1 [71,72,73], Epstein–Barr virus [74], and Kaposi’s sarcoma-associated herpesvirus [75], it may represent an interesting host target for further study in the context of the arenavirus Z protein.

**Figure 2:**
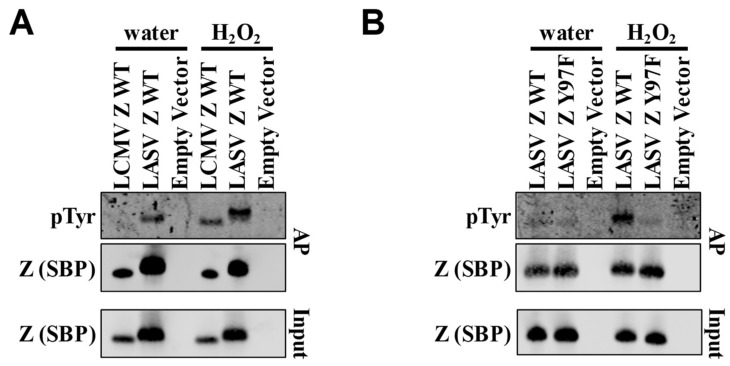
Confirmation of Y97 phosphorylation site in LASV Z. (**A**,**B**) HEK293T cells were transfected with plasmids encoding the indicated streptavidin-binding peptide (SBP)-tagged lymphocytic choriomeningitis virus (LCMV) Z or LASV Z, and two days later streptavidin-coated magnetic beads were used to affinity purify (AP) intracellular Z from cells that had been treated with water or hydrogen peroxide (H_2_O_2_). Levels of phosphotyrosine and Z-SBP in affinity-purified samples (and unpurified cellular input for Z-SBP) were determined by western blotting with anti-phosphotyrosine or anti-SBP antibodies, respectively. Levels of phosphotyrosine are shown for wild-type (WT) LCMV Z and LASV Z (**A**) as well as for WT and phosphosite-mutant (Y97F) LASV Z proteins (**B**). Western blots are representative of five (**A**) or four (**B**) independent experiments.

The Y97 phosphorylation site lies within the PPXY late domain, which is conserved across the majority of Old World arenavirus Z proteins (Figure 1C). Our lab recently showed that the homologous tyrosine residue (Y88) in LCMV Z is also phosphorylated [40]. Treating cells with H_2_O_2_ to block tyrosine phosphatases and permit the accumulation of tyrosines phosphorylated by endogenous kinases demonstrated that both LCMV and LASV Z are phosphorylated on a tyrosine residue (Figure 2A) [76]. This strategy further showed that the phosphotyrosine levels of LASV Z were significantly reduced in the Y97F mutant of LASV Z, indicating that this late domain-embedded tyrosine is likely the major tyrosine phosphorylation site (Figure 2B), similar to what we observed for the PPXY-embedded Y88 in the case of LCMV [40].

**Figure 3:**
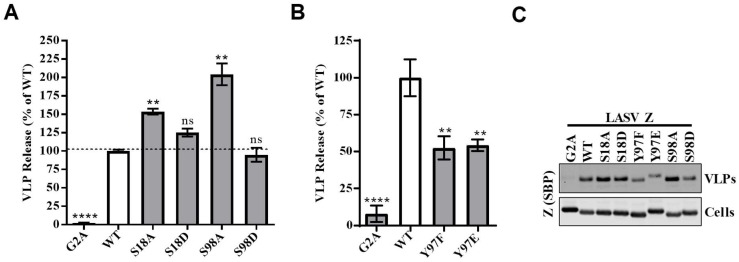
VLP release assay of WT and phosphomutant LASV Z proteins. (**A**–**C**) HEK293T cells were transfected with plasmids encoding the WT LASV Z protein or the LASV Z containing mutations that prevent (S to A; Y to F) or mimic (S to D; Y to E) phosphorylation at the serine phosphorylation sites (**A**) or the tyrosine phosphorylation site (**B**). The glycine-to-alanine mutant (G2A) served as a negative control as it prevents myristoylation of Z, resulting in drastic inhibition of Z’s budding activity. Quantitative, fluorescent western blotting was used to quantify the amount of intracellular and VLP-derived Z protein. A representative western blot of intracellular or VLP-derived SBP-tagged Z protein is shown in (**C**). The VLP release activity was determined by dividing the quantity of Z in VLPs by the quantity of intracellular Z, then normalized to the amount of wild-type Z. The values represent the mean ± standard error of the mean from three (**A**) or four (**B**) independent experiments. Mean values were compared using a one-way ANOVA with the Holm–Sidak’s test for multiple comparisons. (**A**,**B**), n.s. (not significant), ** *p* < 0.01, **** *p* < 0.0001.

A prominent function of the arenavirus Z protein is to drive the release of virus particles. We next sought to determine whether any of the three phosphorylation sites might contribute to the ability of Z to drive virus budding using a VLP release assay (Figure 3A–C). For the phosphoserine residues at S18 or S98, LASV Z protein mutants encoding either an alanine or an aspartic acid substitution at these positions were made in order to prevent or to possibly mimic phosphorylation at the site in question, respectively. Phosphomimetic substitution did not result in a significant change in VLP release for either S18 or S98 (Figure 3A,C). However, substitution with alanine to either phosphoserine site resulted in levels of VLP release that were 1.5- or 2-fold greater than in the WT, suggesting that serine phosphorylation may negatively regulate the release of virus particles (Figure 3A). It is important to note that, in many cases, aspartic acid and glutamic acid do not functionally mimic phosphorylation, particularly when a phosphorylated residue is recognized by an adaptor protein [77]. Our results here could be explained if cellular kinases impinged on viral budding by promoting a phosphoserine-dependent protein–protein interaction, one which cannot be mimicked by the shape or charge afforded by aspartic acid.

For the Y97 phosphosite, phenylalanine and glutamic acid mutants were generated, again to prevent or mimic phosphorylation, respectively. Release of VLPs from cells transfected with LASV Z containing either the non-phosphorylatable (F) or phosphomimetic (E) amino acid at residue 97 was decreased by roughly 50% compared to wild-type Z-containing cells (Figure 3B,C). Given that Y97 lies within the PPXY late domain, which has been previously shown to be required for efficient release of LCMV and LASV VLPs, the results were not surprising [23,32,40] and may indicate a cellular mechanism at play to regulate viral budding. Mutation of the canonical PPXY motif in LASV results in a loss of binding to Nedd4-family E3 ubiquitin ligases, which have been implicated in the release of several other viruses that have a PPXY domain in their matrix protein [24,36]. However, in earlier work with LCMV, despite a reduction in VLP release, disruption of the PPXY late domain did not block the efficient release of infectious virus particles but rather that of defective interfering (DI) particles [40]. Furthermore, greater levels of DI particles were released with recombinant LCMV containing a glutamic acid substitution at its Y88 phosphorylation site relative to the Y88F-mutant virus, suggesting that phosphorylation may positively regulate the release of DI particles [40]. These data raise the intriguing question of whether a similar phenomenon (e.g., whereby phosphorylation of the PPXY late domain regulates the release of a specific class of viral particles) may be occurring with the LASV Z protein. There is currently little data confirming the presence of LASV DI particles. However, it is thought that most animal viruses produce some level of DI particles, and some arenaviruses in particular are known to produce significant levels of DI particles [78,79]. More specifically, a high multiplicity of infection with LASV virus yields lower infectious titers and higher ratios of viral genomic RNA to particles compared with low-multiplicity infections [80]. These findings are consistent with a virus that produces appreciable levels of DI particles [81,82]. 

The discovery of these phosphorylation sites opens up several new avenues for inquiry, including identifying the host kinases involved as well as any phosphosite-binding proteins that may regulate Z protein function. Kinase prediction algorithms could be used to narrow the possible candidate kinases, but empirical screens will be required to determine the kinase(s) that contribute most substantially to Z phosphorylation. Kinase identification for the LASV Z Y97 phosphosite may be aided by evidence in the literature implicating Src family or Abl tyrosine kinases in the phosphorylation of PPXY domains in other proteins—specifically, the Ebola virus matrix protein and the cellular proteins dystroglycan and IFITM3 [46,83,84]. It has also been shown for dystroglycan and IFITM3 that phosphorylation of PPXY motifs can regulate the binding of proteins that contain SH2 domains as well as of the Nedd4-family ubiquitin ligases that bind PPXY domains through their WW domain [83,84,85]. It is plausible that Old World mammarenavirus Z proteins are regulated in a similar fashion. It will also be important to identify phosphoserine-specific binding proteins (e.g., perhaps Pin1) or proteins containing other phosphoserine-binding domains in order to understand the mechanism by which serine phosphorylation regulates Z protein function [66]. Furthermore, it will be important to determine whether the S18 phosphorylation site or other residues in the N-terminal domain, that are more highly conserved among pathogenic arenaviruses, are involved in the inhibition of RLR signaling and macrophage activation [25,59]. Finally, the clustering of two phosphorylation sites at the C-terminus of the LASV Z protein, spanning two distinct WW domain-binding motifs, contributes to the idea that this region of Z and its corresponding functions may be intricately regulated by a network of distinct host protein partners.

## 3. Materials and Methods

### 3.1. Cells and Plasmids

Human embryonic kidney cells (HEK-293T/17, CRL-11268), purchased from American Type Culture Collection (Manassas, VA, USA), were cultured in Dulbecco’s Modified Eagle Medium (DMEM) (11965-092) supplemented with 10% fetal bovine serum (FBS) (16140-071) and 1% of penicillin/streptomycin (15140-122), MEM Non-Essential Amino Acids Solution (11140-050), HEPES Buffer Solution (15630-130), and GlutaMAX (35050-061) purchased from Thermo Fisher Scientific (Carlsbad, CA, USA). 

Plasmid LASV Z HA-BAP expresses the LASV Z gene from strain Josiah (GenBank #HQ688675.1) with a C-terminal hemagglutinin (HA) affinity tag followed by a tobacco etch virus cleavage site and a biotinylation acceptor peptide in a modified pCAGGS expression vector, as previously described [86,87]. Plasmid LASV Z WT SBP is comprised of the LASV Z gene C-terminally fused to a six-amino acid linker (AAGGGG) followed by the streptavidin-binding peptide (SBP) affinity tag in a modified pCAGGS vector, as previously described [88]. Genes encoding point mutants of LASV Z (S18A, S18D, Y97F, Y97E, S98A, and S98D) were synthesized and subcloned into the LASV Z WT SBP vector by BioBasic, Inc. (Markham, ON, Canada). Plasmids expressing the Armstrong 53b strain of LCMV Z (GenBank #AY847351.1) with a C-terminal SBP tag have been described previously [40], as has the plasmid encoding the biotin ligase (BirA) [87].

### 3.2. Identification of Phosphorylation Sites by Mass Spectrometry

To identify potential phosphorylation sites in LASV Z, 1 × 10^6^ HEK293T cells/well were seeded into two six-well plates. After 24 h, each well was transfected with 100 μL DMEM containing 1 μg of LASV Z HA-BAP, 1 μg of plasmid BirA, and 10 μg of polyethyleneinimine (23966, Polysciences, Inc., Warrington, PA, USA). After 48 h, the VLP-containing culture media was collected and clarified by centrifugation, then a solution of 1x Triton lysis buffer (0.5% NP40, 1% Triton X-100 (BP151-100, Fisher Scientific), 140 mM NaCl, 25 mM Tris HCl) with protease (04693159001, Roche, Indianapolis, IN, USA) and phosphatase inhibitor cocktails (4906837001, Roche) was added to lyse the VLPs. Biotin-modified LASV Z protein was affinity purified by incubating the VLP lysate with Dynabeads MyOne Streptavidin T1 beads (65602, Thermo Fisher Scientific) for 2 h at 4 °C while rotating. Following incubation, the beads were washed with 1x Triton lysis buffer and then were eluted in 4x Laemmli sample buffer (250 mM Tris-HCl, 40% glycerol, 8% sodium dodecyl sulfate, and 0.04% bromophenol blue) diluted to 1x in Triton lysis buffer with a final concentration of 5% 2-mercaptoethanol by heating at 100 °C for 10 min. The protein eluate was subjected to electrophoresis on a 4–20% Tris-glycine polyacrylamide gel (EC60285BOX, Invitrogen, Carlsbad, CA, USA). This gel was then stained overnight with Coomassie (40% methanol, 20% acetic acid, and 0.1% Brilliant Blue R (B7920, Sigma-Aldrich, St. Louis, MI, USA) followed by de-staining with a solution of 30% methanol and 10% acetic acid and was imaged on a Canon Canoscan 8800F scanner. The region of the gel lane containing the Z protein was excised and either directly reduced and alkylated with iodoacetamide or not, prior to being subjected to in-gel digestion with a solution of sequencing-grade modified trypsin (V5111, Promega, Madison, WI, USA) or a mixture of trypsin and sequencing-grade chymotrypsin (V1061, Promega), as described previously [40,51]. The resultant peptides were extracted from the gel slice using 2.5% formic acid in 50% acetonitrile and centrifugation. This supernatant was collected, and the gel slice was further dehydrated by incubating with 100% acetonitrile, centrifuging, and collecting the supernatant twice. The solvent was evaporated using a vacuum centrifuge at 37 °C, and the peptides were resuspended in 2.5% acetonitrile and 2.5% formic acid. Liquid chromatography was conducted using a microcapillary column packed with 12 cm Magic C18, 200 Å, 5 μm material (PM5/66100/00, Michrom Bioresources, Auburn, CA, USA) using a MicroAS autosampler (Thermo Scientific, Pittsburgh, PA, USA). The peptides were eluted with a gradient of 5−35% acetonitrile (0.15% formic acid) using a Surveyor Pump Plus HPLC (Thermo Scientific) over 40 min after a 15 min isocratic loading at 2.5% acetonitrile and 0.15% formic acid. Data were acquired in both a stand-alone linear ion trap mass spectrometer (LTQ-XL) and a linear ion trap–orbitrap (LTQ–Orbitrap) hybrid mass spectrometer (both instruments from Thermo Scientific). Ten MS/MS scans in the LTQ followed each linear ion trap or orbitrap survey scan over the entire run. A concatenated database of the LASV Z protein sequence including affinity tags in forward and reverse orientation was queried with SEQUEST software with no enzyme requirement and a 20 PPM precursor mass tolerance (or 2 Da for data collected in the stand-alone LTQ). The following differential modifications were allowed: +79.96633 Da for phosphorylation of serine, threonine, and tyrosine; +15.99492 Da for methionine oxidation; and either 71.0371 Da for cysteine acrylamidation or +57.02146 for cysteine carbamidomethylation. 

### 3.3. Detection of Phosphoproteins by Western Blotting

In order to confirm the presence of phosphorylated LASV Z protein, 5 × 10^5^ HEK293T cells per well were seeded in six-well plates and then transfected 24 h later with 2 μg of the corresponding Z protein plasmids using 8 μg of 1 mg/mL polyethyleneinimine. Two days after transfection, the cells were treated with 8.8 mM hydrogen peroxide, or water as a control, for 20 min (min) for phosphotyrosine detection. The cells were then lysed with 1x Triton lysis buffer containing both protease and phosphatase inhibitor cocktails. SBP-tagged Z was affinity purified by incubating with streptavidin-coated magnetic beads for 2 h, followed by elution in 2x Laemmli sample buffer (125 mM Tris-HCl, 20% glycerol, 4% sodium dodecyl sulfate, and 0.02% bromophenol blue) with 5% 2-mercaptoethanol by heating at 100 °C for 10 min. The purified samples or cell input prepared in Laemmli sample buffer were separated by electrophoresis using NuPAGE 4–12% Bis-Tris gels (Thermo Fisher Scientific) with MES buffer (B000202, Thermo Fisher Scientific). Western blotting was carried out using nitrocellulose iBlot 2 gel transfer stacks (IB23001, Thermo Fisher Scientific) and the Invitrogen iBlot 2 transfer apparatus. Membranes for phosphotyrosine detection were blocked for 1 h in protein-free blocking buffer (37572, Thermo Fisher Scientific), and then a mouse anti-phosphotyrosine antibody (clone 4G10, Millipore) diluted (0.2 μg/mL) in protein-free blocking buffer was added to the membrane and incubated overnight at room temperature. The membrane was washed five times with Tris-buffered saline containing 0.1% Tween 20 (BP337, Fisher Scientific, Pittsburgh, PA, USA) (TBST), incubated for 1 h with goat anti-mouse 800 secondary antibody (926-32210, Licor, Lincoln, NE, USA) diluted 1:20,000 in 5% milk, 0.2% Tween 20 (BP337, Fisher Scientific), and 0.02% sodium dodecyl sulfate in phosphate buffered saline (PBS), and then imaged using a Licor Odyssey CLx imaging system. Immunoblotting for the Z protein was conducted by incubating the membrane with an anti-SBP tag antibody (MAB10764, Millipore, Billerica, MA, USA) diluted 1:10,000 and an anti-mouse 800 secondary antibody (926-32210, Licor) diluted 1:3000 in iBind fluorescent detection solution (SLF2019, Thermo Fisher Scientific) in an iBind Flex western device.

### 3.4. Virus-Like Particle Release Assay

To measure VLP release, 2 × 10^5^ HEK293T cells per well were seeded in 12-well plates and then transfected 24 h later with 0.8 μg of the designated SBP-tagged LASV Z protein-expressing plasmids, using 3.2 μg of polyethyleneinimine. One day later, the VLP-containing cell culture media was collected, clarified by centrifugation, and then mixed with 10x Triton lysis buffer with a protease inhibitor cocktail to a final concentration of 1x. The cells were scraped into phosphate-buffered saline, pelleted by centrifugation, and then lysed with 1x Triton lysis buffer containing a protease inhibitor cocktail. The samples were subjected to SDS-PAGE and western blotting as described above. The SBP-tagged Z protein was detected using an anti-SBP tag antibody (MAB10764, Millipore), diluted 1:10,000 in 5% nonfat milk and 0.2% Tween 20 in PBS, and the anti-mouse 800 secondary antibody (926-32210, Licor), diluted 1:20,000 in 5% nonfat milk, 0.02% sodium dodecyl sulfate, and 0.2% Tween 20 in PBS. In order to determine the percent VLP release, the value of each Z protein band on a particular blot was first divided by the sum of the values of all the Z protein bands on that particular blot (normalization by summation as described in [89]). This normalized Z protein quantity in VLPs was divided by the normalized Z quantity in cells for each mutant, and these values were then normalized to the corresponding quotient of Z WT, as described previously [40]. A one-way ANOVA with Holm–Sidak’s test for multiple comparisons in GraphPad Prism software was used to analyze differences in VLP release.

## Supplementary Materials

The following are available online at http://www.mdpi.com/2076-0817/7/4/97/s1. Figure S1: Mass spectra for identification of the Y97 and S98 phosphorylation sites in LASV Z, Figure S2: Mass spectra for identification of the S18 phosphorylation site in LASV Z.
