# Supplementary material for: Host-Driven Phosphorylation Appears to Regulate the Budding Activity of the Lassa Virus Matrix Protein
Source: Pathogens. 2018 Dec 9;7(4):97. doi: 10.3390/pathogens7040097 (PMC6313517; doi:10.3390/pathogens7040097)
Supplement: Supplementary file 1 [file pathogens-07-00097-s001.pdf]

A

LASV pY97 ; *z*=3 ; XCorr = 4.6

Sequence: LRPSAAPTAPPTGAADSIRPPY#SPDPAFLY

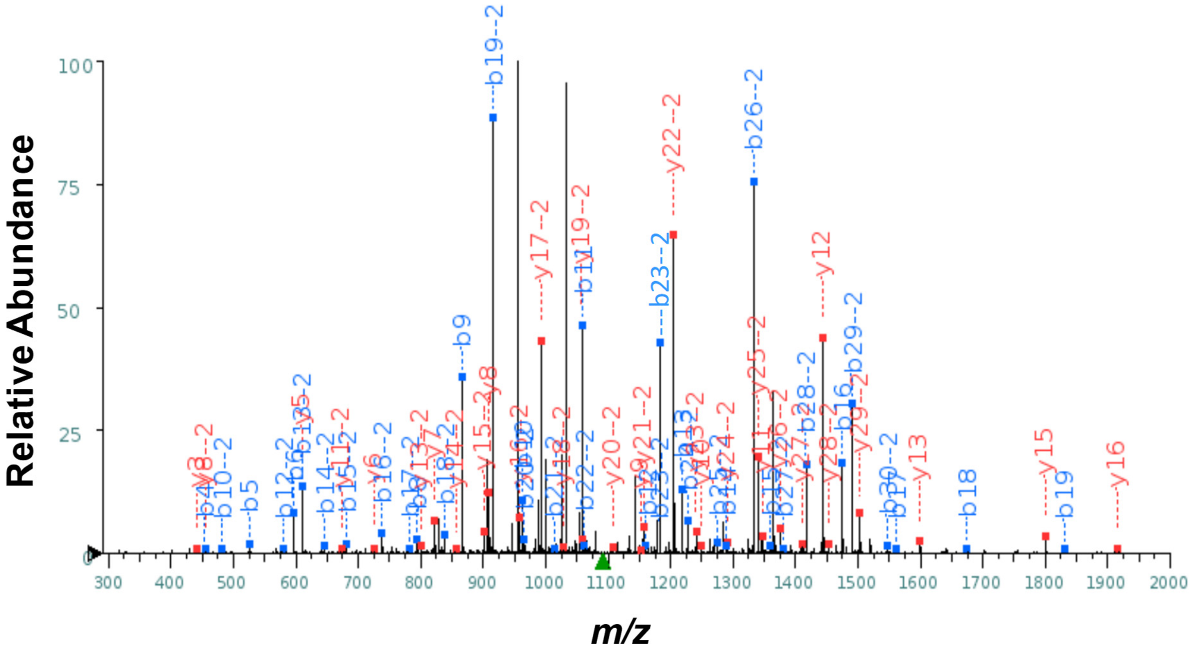

Fragment Ions *z*=1

Seq	#	b: Δ Error	b	y	y: Δ Error	+1
L	1	---	114.091	---	---	31
R	2	---	270.192	3160.514	---	30
P	3	---	367.245	3004.413	---	29
S	4	196.209	454.277	2907.360	---	28
A	5	326.564	525.314	2820.328	---	27
A	6	104.742	596.351	2749.291	---	26
P	7	---	693.404	2678.254	---	25
T	8	63.674	794.452	2581.201	---	24
A	9	54.847	865.489	2480.153	---	23
P	10	-1032.797	962.542	2409.116	---	22
P	11	-23.455	1059.595	2312.063	---	21
T	12	94.315	1160.642	2215.011	---	20
G	13	211.381	1217.664	2113.963	---	19
A	14	-28.611	1288.701	2056.942	---	18
A	15	-5.214	1359.738	1985.904	---	17
D	16	-256.396	1474.765	1914.867	42.302	16
S	17	-53.556	1561.797	1799.840	-20.325	15
I	18	-81.185	1674.881	1712.808	---	14
R	19	168.237	1830.982	1599.724	-54.994	13
P	20	---	1928.035	1443.623	-75.123	12
P	21	---	2025.088	1346.570	56.192	11
P	22	---	2122.140	1249.518	-162.087	10
Y#	23	---	2365.170	1152.465	306.905	9
S	24	---	2452.202	909.435	-17.404	8
P	25	---	2549.255	822.403	90.938	7
D	26	---	2664.282	725.350	322.925	6
P	27	---	2761.335	610.324	-10.265	5
A	28	---	2832.372	513.271	---	4
F	29	---	2979.440	442.234	307.599	3
L	30	---	3092.524	295.165	---	2
Y	31	---	---	182.081	---	1

Fragment Ions *z*=2

Seq	#	b: Δ Error	b	y	y: Δ Error	+1
L	1	---	57.549	---	---	31
R	2	---	135.600	1580.761	---	30
P	3	---	184.126	1502.710	234.080	29
S	4	---	227.642	1454.184	-11.470	28
A	5	---	263.161	1410.668	8.355	27
A	6	---	298.679	1375.149	155.283	26
P	7	---	347.206	1339.631	199.754	25
T	8	---	397.730	1291.104	-157.095	24
A	9	---	433.248	1240.580	494.106	23
P	10	450.496	481.775	1205.062	212.378	22
P	11	---	530.301	1156.535	206.952	21
T	12	370.306	580.825	1108.009	-11.650	20
G	13	1608.629	609.335	1057.485	253.147	19
A	14	461.048	644.854	1028.974	-597.738	18
A	15	208.467	680.373	993.456	736.640	17
D	16	-74.235	737.886	957.937	-871.554	16
S	17	207.824	781.402	900.424	184.999	15
I	18	-66.902	837.944	856.908	-723.901	14
R	19	84.701	915.995	800.366	159.260	13
P	20	399.963	964.521	722.315	---	12
P	21	102.701	1013.047	673.789	-662.135	11
P	22	-346.784	1061.574	625.262	---	10
Y#	23	242.804	1183.089	576.736	---	9
S	24	209.901	1226.605	455.221	-1881.465	8
P	25	303.519	1275.131	411.705	---	7
D	26	215.225	1332.645	363.179	---	6
P	27	140.684	1381.171	305.665	---	5
A	28	169.803	1416.689	257.139	---	4
F	29	209.473	1490.224	221.620	---	3
L	30	236.106	1546.766	148.086	---	2
Y	31	---	---	91.544	---	1

B

LASV Y97, S98 ; *z*=3 ; XCorr = 2.6

Sequence: LRPSAAPTAPPTGAADSIRPPYSPDPAFLY

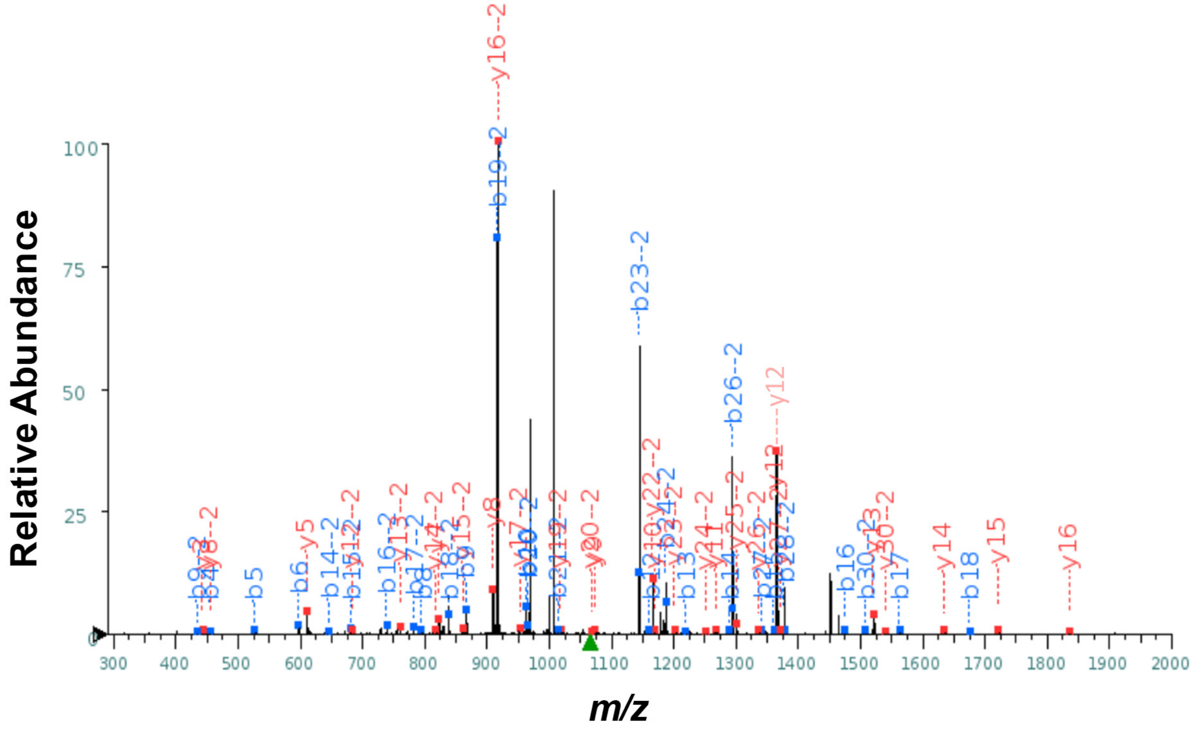

Fragment Ions *z*=1

Seq	#	b: Δ Error	b	y	y: Δ Error	+1
L	1	---	114.091	---	---	31
R	2	---	270.192	3080.548	---	30
P	3	---	367.245	2924.447	---	29
S	4	548.565	**454.277**	2827.394	---	28
A	5	470.638	**525.314**	2740.362	---	27
A	6	168.589	**596.351**	2669.325	---	26
P	7	---	693.404	2598.287	---	25
T	8	156.998	**794.452**	2501.235	---	24
A	9	88.551	**865.489**	2400.187	---	23
P	10	-52.805	**962.542**	2329.150	---	22
P	11	---	1059.595	2232.097	---	21
T	12	-643.576	**1160.642**	2135.044	---	20
G	13	269.799	**1217.664**	2033.997	---	19
A	14	735.551	**1288.701**	1976.975	---	18
A	15	559.426	**1359.738**	1905.938	---	17
D	16	-62.315	**1474.765**	**1834.901**	511.050	16
S	17	525.416	**1561.797**	**1719.874**	-8.198	15
I	18	59.555	**1674.881**	**1632.842**	609.506	14
R	19	---	1830.982	**1519.758**	603.781	13
P	20	---	1928.035	**1363.657**	688.701	12
P	21	---	2025.088	**1266.604**	731.063	11
P	22	---	2122.140	**1169.551**	420.169	10
Y	23	---	2285.204	**1072.499**	-18.831	9
S	24	---	2372.236	**909.435**	-41.566	8
P	25	---	2469.288	**822.403**	40.775	7
D	26	---	2584.315	725.350	---	6
P	27	---	2681.368	**610.324**	55.835	5
A	28	---	2752.405	513.271	---	4
F	29	---	2899.474	**442.234**	2226.869	3
L	30	---	3012.558	295.165	---	2
Y	31	---	---	182.081	---	1

Fragment Ions *z*=2

Seq	#	b: Δ Error	b	y	y: Δ Error	+1
L	1	---	57.549	---	---	31
R	2	---	135.600	1540.777	---	30
P	3	---	184.126	1462.727	---	29
S	4	---	227.642	1414.201	---	28
A	5	---	263.161	**1370.684**	-644.336	27
A	6	---	298.679	**1335.166**	289.709	26
P	7	---	347.206	**1299.647**	695.381	25
T	8	---	397.730	**1251.121**	632.606	24
A	9	---	433.248	**1200.597**	247.525	23
P	10	-194.689	**481.775**	**1165.079**	701.506	22
P	11	-1861.696	**530.301**	1116.552	---	21
T	12	---	580.825	1068.026	---	20
G	13	---	609.335	**1017.502**	-495.047	19
A	14	616.863	**644.854**	**988.991**	-617.953	18
A	15	575.715	**680.373**	**953.473**	-414.394	17
D	16	643.664	**737.886**	**917.954**	-180.808	16
S	17	972.319	**781.402**	**860.441**	372.829	15
I	18	500.997	**837.944**	**816.925**	825.966	14
R	19	504.373	**915.995**	**760.383**	468.064	13
P	20	147.107	**964.521**	**682.332**	-962.412	12
P	21	-965.230	**1013.047**	633.806	---	11
P	22	---	1061.574	585.279	---	10
Y	23	743.108	**1143.105**	536.753	---	9
S	24	344.574	**1186.621**	**455.221**	497.281	8
P	25	214.176	**1235.148**	411.705	---	7
D	26	698.150	**1292.661**	363.179	---	6
P	27	-334.407	**1341.188**	305.665	---	5
A	28	336.301	**1376.706**	257.139	---	4
F	29	---	1450.240	221.620	---	3
L	30	---	1506.783	148.086	---	2
Y	31	---	---	91.544	---	1

C

LASV pS98 ; *z*=3 ; XCorr = 3.2

Sequence: LRPSAAPTAPPTGAADSIRPPYS#PDPAFLY

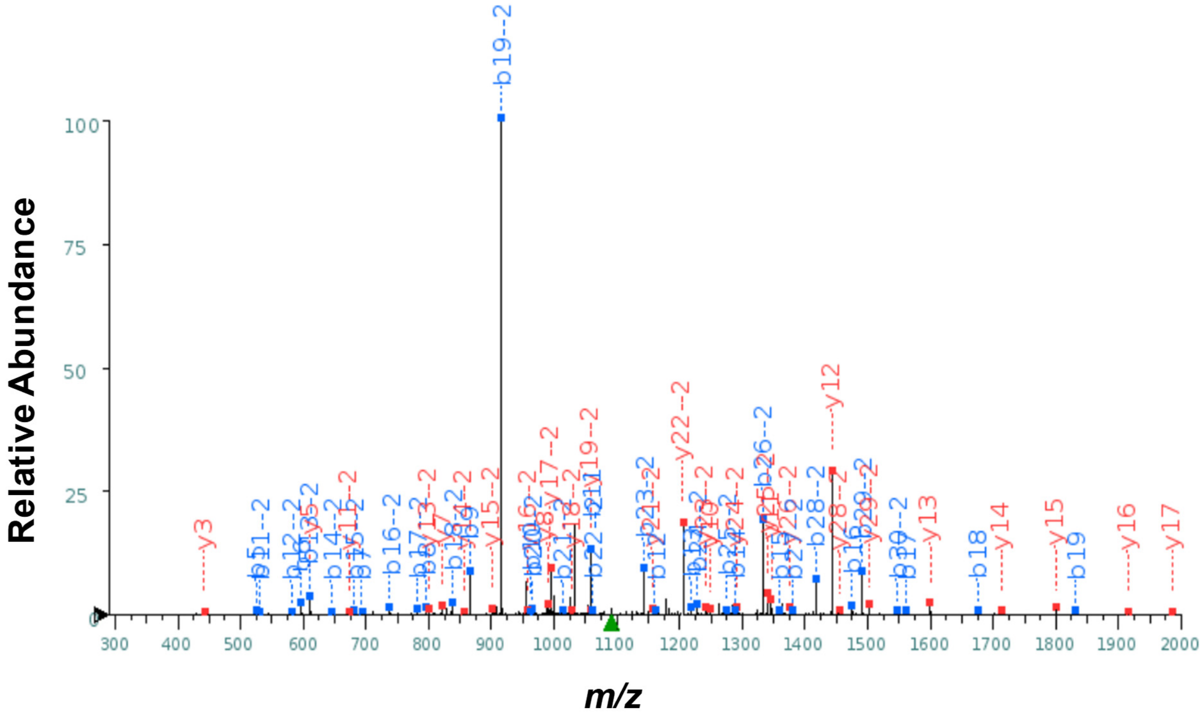

Fragment Ions *z*=1

Seq	#	b: Δ Error	b	y	y: Δ Error	+1
L	1	---	114.091	---	---	31
R	2	---	270.192	3160.514	---	30
P	3	---	367.245	3004.413	---	29
S	4	---	454.277	2907.360	---	28
A	5	153.760	**525.314**	2820.328	---	27
A	6	88.881	**596.351**	2749.291	---	26
P	7	318.102	**693.404**	2678.254	---	25
T	8	-1.701	**794.452**	2581.201	---	24
A	9	46.949	**865.489**	2480.153	---	23
P	10	42.312	**962.542**	2409.116	---	22
P	11	-166.336	**1059.595**	2312.063	---	21
T	12	633.889	**1160.642**	2215.011	---	20
G	13	164.683	**1217.664**	2113.963	---	19
A	14	101.435	**1288.701**	2056.942	---	18
A	15	253.004	**1359.738**	**1985.904**	29.354	17
D	16	-60.990	**1474.765**	**1914.867**	410.157	16
S	17	-45.895	**1561.797**	**1799.840**	-34.569	15
I	18	-26.226	**1674.881**	**1712.808**	-57.967	14
R	19	-17.476	**1830.982**	**1599.724**	-104.754	13
P	20	---	1928.035	**1443.623**	-64.298	12
P	21	---	2025.088	**1346.570**	-726.303	11
P	22	---	2122.140	**1249.518**	-148.406	10
Y	23	---	2285.204	1152.465	---	9
S#	24	---	2452.202	**989.402**	-65.374	8
P	25	---	2549.255	**822.403**	209.648	7
D	26	---	2664.282	725.350	---	6
P	27	---	2761.335	**610.324**	-5.965	5
A	28	---	2832.372	513.271	---	4
F	29	---	2979.440	**442.234**	178.962	3
L	30	---	3092.524	295.165	---	2
Y	31	---	---	182.081	---	1

Fragment Ions *z*=2

Seq	#	b: Δ Error	b	y	y: Δ Error	+1
L	1	---	57.549	---	---	31
R	2	---	135.600	1580.761	---	30
P	3	---	184.126	**1502.710**	374.528	29
S	4	---	227.642	**1454.184**	-275.215	28
A	5	---	263.161	1410.668	---	27
A	6	---	298.679	**1375.149**	39.018	26
P	7	---	347.206	**1339.631**	-106.662	25
T	8	---	397.730	**1291.104**	-60.258	24
A	9	---	433.248	**1240.580**	-6.199	23
P	10	---	481.775	**1205.062**	149.596	22
P	11	---	530.301	**1156.535**	-225.910	21
T	12	-157.312	**580.825**	1108.009	---	20
G	13	---	609.335	1057.485	---	19
A	14	---	644.854	**1028.974**	174.059	18
A	15	---	680.373	**993.456**	633.689	17
D	16	36.113	**737.886**	**957.937**	122.764	16
S	17	---	781.402	**900.424**	407.473	15
I	18	183.199	**837.944**	856.908	---	14
R	19	225.519	**915.995**	**800.366**	450.012	13
P	20	668.996	**964.521**	**722.315**	-1077.593	12
P	21	652.601	**1013.047**	**673.789**	-579.510	11
P	22	-227.010	**1061.574**	625.262	---	10
Y	23	-26.711	**1143.105**	576.736	---	9
S#	24	149.117	**1226.605**	495.204	---	8
P	25	-120.782	**1275.131**	411.705	---	7
D	26	176.035	**1332.645**	363.179	---	6
P	27	-50.115	**1381.171**	305.665	---	5
A	28	226.133	**1416.689**	257.139	---	4
F	29	180.896	**1490.224**	221.620	---	3
L	30	288.166	**1546.766**	148.086	---	2
Y	31	---	---	91.544	---	1

Supplemental Figure S1. Mass spectrometry-based identification of Y97 and S98 phosphorylation sites in LASV Z. Low energy collision-induced dissociation MS/MS spectra of the doubly-digested (trypsin and chymotrypsin) peptide ions harboring (A) phosphorylated Y97, (B) unphosphorylated Y97 and S98, or (C) phosphorylated S98 on LASV Z protein. Tables indicate the theoretical *m/z* values for the fragment (1+ and 2+) ions. Red and blue font indicate observed values. S# and Y# denote phosphoserine and phosphotyrosine, respectively. Peptides include five amino acids (DPAFLY) of the affinity tag at the C-termini. Green triangles indicate the *m/z* values of the precursors. Precursor charge states and SEQUEST XCorr values are indicated.

A

LASV pS18 ; *z*=2 ; XCorr = 1.5

Sequence: AS#LIPDATHLGPQFCK

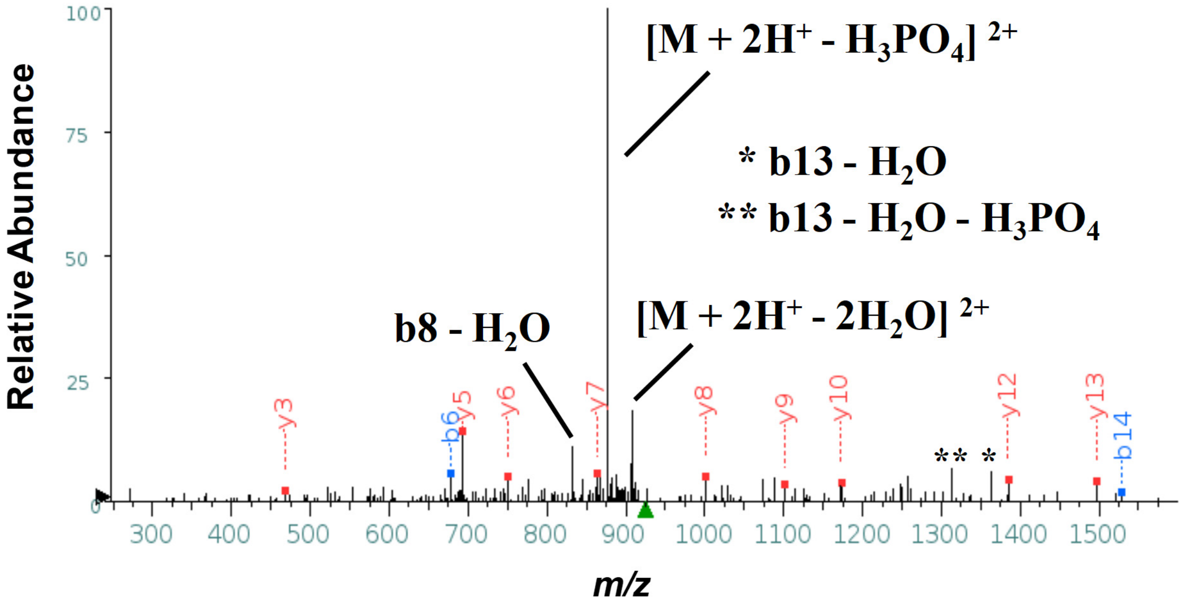

Fragment Ions *z*=1

Seq	#	b: Δ Error	b	y	y: Δ Error	+1
A	1	---	72.044	---	---	16
S#	2	---	239.043	1777.834	---	15
L	3	---	352.127	1610.836	---	14
I	4	---	465.211	**1497.752**	-496.616	13
P	5	---	562.264	**1384.668**	182.667	12
D	6	1393.249	**677.291**	1287.615	---	11
A	7	---	748.328	**1172.588**	428.448	10
T	8	---	849.375	**1101.551**	21.875	9
H	9	---	986.434	**1000.503**	758.684	8
L	10	---	1099.518	**863.444**	96.634	7
G	11	---	1156.540	**750.360**	28.427	6
P	12	---	1253.593	**693.339**	-524.573	5
Q	13	---	1381.651	596.286	---	4
F	14	85.616	**1528.720**	**468.228**	100.280	3
C@	15	---	1702.766	321.159	---	2
K	16	---	---	147.113	---	1

B

LASV S18 ; *z*=2 ; XCorr = 4.1

Sequence: ASLIPDATHLGPQFCK

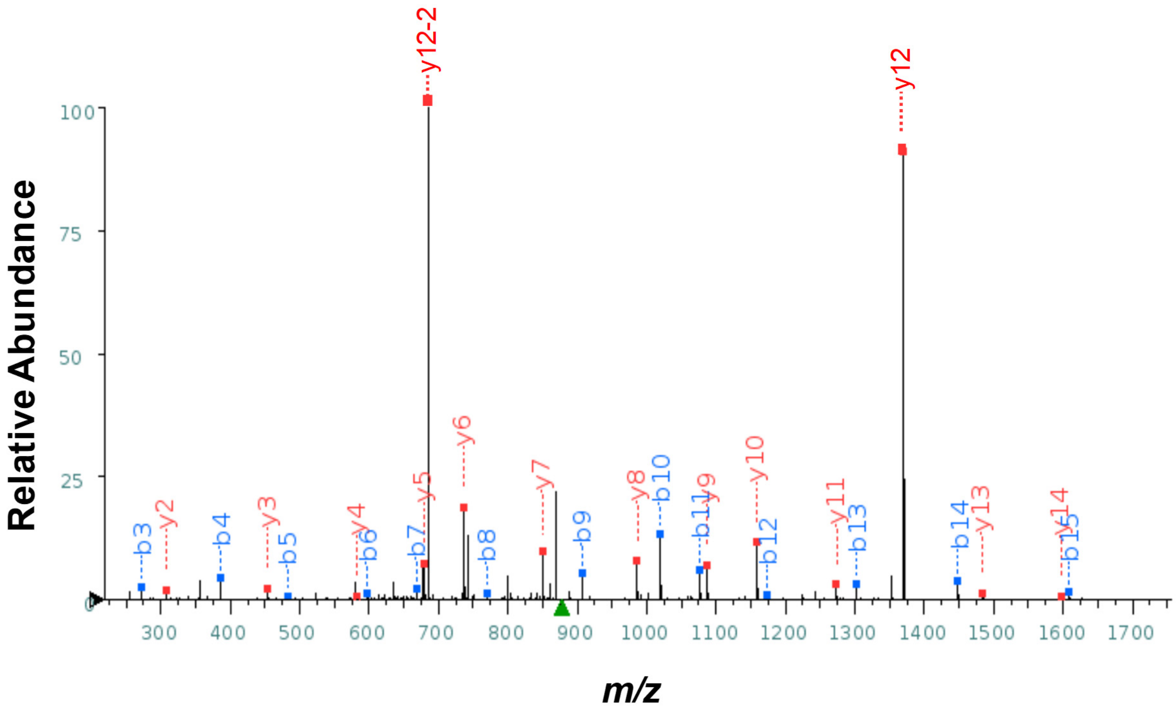

Fragment Ions *z*=1

Seq	#	b: Δ Error	b	y	y: Δ Error	+1
A	1	---	72.044	---	---	16
S	2	---	159.076	1683.852	---	15
L	3	-601.684	272.160	1596.820	-17.786	14
I	4	-207.374	385.245	1483.736	-59.569	13
P	5	282.239	482.297	1370.652	-25.058	12
D	6	-133.115	597.324	1273.599	-37.488	11
A	7	-210.962	668.361	1158.572	-18.270	10
T	8	114.948	769.409	1087.535	2.766	9
H	9	-9.831	906.468	986.488	-9.191	8
L	10	-49.170	1019.552	849.429	-0.312	7
G	11	-16.213	1076.573	736.345	96.471	6
P	12	108.448	1173.626	679.323	131.125	5
Q	13	-49.902	1301.685	582.270	464.893	4
F	14	-78.834	1448.753	454.212	87.046	3
C	15	-25.464	1608.784	307.143	214.616	2
K	16	---	---	147.113	---	1

Supplemental Figure S2. Mass spectrometry-based identification of S18 phosphorylation site in LASV Z. Low energy collision-induced dissociation MS/MS spectra of the tryptic peptide ions harboring (A) phosphorylated or (B) unphosphorylated serine 18 on the LASV Z protein. Tables indicate the theoretical *m/z* values for the fragment (1+) ions. Red and blue font indicate observed values. S# denotes phosphoserine. Green triangles indicate the *m/z* values of the precursors. Spectra include cysteine with a mass increase of 71 Da for acrylamidation or 57 for carbamidomethylation. Major peaks unexplained by y- and b-type ions are labeled in A. Precursor charge states and SEQUEST XCorr values are indicated.
